# Reducing Length of Hospital Stay Does Not Increase Readmission Rates in Early-Stage Gastric, Colon, and Lung Cancer Surgical Cases in Japanese Acute Care Hospitals

**DOI:** 10.1371/journal.pone.0166269

**Published:** 2016-11-10

**Authors:** Susumu Kunisawa, Kiyohide Fushimi, Yuichi Imanaka

**Affiliations:** 1 Department of Healthcare Economics and Quality Management, Graduate School of Medicine, Kyoto University, Kyoto, Japan; 2 Department of Health Policy and Informatics, Tokyo Medical and Dental University, Tokyo, Japan; Osaka University Graduate School of Medicine, JAPAN

## Abstract

**Background:**

The Japanese government has worked to reduce the length of hospital stay by introducing a per-diem hospital payment system that financially incentivizes the timely discharge of patients. However, there are concerns that excessively reducing length of stay may reduce healthcare quality, such as increasing readmission rates. The objective of this study was to investigate the temporal changes in length of stay and readmission rates as quality indicators in Japanese acute care hospitals.

**Methods:**

We used an administrative claims database under the Diagnosis Procedure Combination Per-Diem Payment System for Japanese hospitals. Using this database, we selected hospitals that provided data continuously from July 2010 to March 2014 to enable analyses of temporal changes in length of stay and readmission rates. We selected stage I (T1N0M0) gastric, colon, and lung cancer surgical patients who had been discharged alive from the index hospitalization. The outcome measures were length of stay during the index hospitalization and unplanned emergency readmissions within 30 days after discharge.

**Results:**

From among 804 hospitals, we analyzed 42,585, 15,467, and 40,156 surgical patients for gastric, colon, and lung cancer, respectively. Length of stay was reduced by approximately 0.5 days per year. In contrast, readmission rates were generally stable at approximately 2% or had decreased slightly over the 4-year period.

**Conclusions:**

In early-stage gastric, colon, and lung cancer surgical patients in Japan, reductions in length of stay did not result in increased readmission rates.

## Introduction

Due to rising healthcare costs, population aging, and shifting patient preferences, there is a need to increase the quality, efficiency, and value of health care. Reducing the length of stay (LOS) in hospitalized patients is a major target for improving healthcare quality and reducing unnecessary costs. In particular, the reduction of LOS is strongly advocated in Japan as statistics have shown that hospitalizations in Japanese hospitals are longer than those in other developed countries.[1]

LOS durations in acute care hospitals have decreased over the past decade in Japan (OECD: Length of hospital stay https://data.oecd.org/healthcare/length-of-hospital-stay.htm). The introduction of the Diagnosis Procedure Combination Per-Diem Payment System (DPC/PDPS) for acute care hospitals in 2003 is likely to be a major cause for this reduction. Under this system, hospital reimbursements from insurers for individual patients are reduced as LOS increases. The Japanese Ministry of Health, Labour and Welfare (MHLW) calculates the average LOS duration and cost for each DPC group (comprising patients with similar diagnoses and procedures) every two years, and specifies higher daily reimbursements in the early stages of hospitalization, moderate daily reimbursements for average LOS durations, and lower daily reimbursements for hospitalizations that exceed average LOS durations. In this way, the payment system disincentivizes the protracted hospitalization of patients. Furthermore, the MHLW has implemented another measure to encourage the appropriate reduction of excessive LOS by applying “adjustment factors” (such as LOS and general patient severity) in the calculation of bonus reimbursements. Each DPC/PDPS hospital is evaluated every year according to these adjustment factors, and a hospital-specific reimbursement magnification ratio is calculated. The implementation of this measure may have influenced other aspects of hospital management. The analyzed data, such as the mean LOS for each hospital, is available on the MHLW website (http://www.mhlw.go.jp/stf/shingi/shingi-chuo.html?tid=128164). As a result, hospital managers may have become more aware of the indicators used for performance measures and their effects on reimbursements.

Within the drive to reduce LOS, there are concerns that excessive reductions may worsen the quality of health care. The MHLW’s Central Social Insurance Medical Council has noted increases in “unexpected readmission rates”[2,3], and indicated that this may be due to the more drastic reduction in LOS in DPC/PDPS hospitals relative to hospitals reimbursed under the conventional pay-for-performance system. However, there are several problems in the unexpected readmission rates reported by the council.[2,3] First, the definition of “unexpected” is ambiguous, as it does not address the specific criteria for what constitutes an expected readmission. Therefore, different interpretations of this definition may give rise to variations in reported data across hospitals. Another issue is that the council conducts analyses based on the causes of readmission without focusing on prior hospitalizations. As a consequence, the council has not examined patient cohorts that were hospitalized for specific diseases with subsequent readmission (for various causes) as an outcome.

Previous studies have investigated the causal associations between LOS and subsequent readmission, but have not produced any definitive conclusions. Several studies have suggested a certain degree of association between the two measures because both LOS and readmission rates improved when hospitalizations were managed using systems such as clinical pathways.[4–6] Another study has reported a relationship between low LOS and high readmission rates.[7] Similarly, Otsubo and Imanaka reported high readmission rates and short LOS for acute myocardial infarction (AMI) patients across various countries.[8] Hamada et al. investigated the increased readmission rate of AMI patients in Japan before and after the introduction of the DPC/PDPS.[9] However, little is known about the association between these two measures in cancer patients in Japan.

In this study, we investigate recent trends in LOS and readmissions in early-stage cancer surgical patients in Japanese acute care hospitals using a large-scale administrative claims database approximately 10 years after the DPC/PDPS was introduced.

## Methods

This study used a database comprising DPC administrative claims data for patients hospitalized between July 2010 and March 2014 (i.e., covering a portion of fiscal year 2010 to the end of fiscal year 2013, where a fiscal year in Japan spans from April to March of the following year). The data were collected by the DPC Study Group, which is funded by the MHLW. Approximately 80% of all DPC hospitals voluntarily participate in the DPC Study, and collectively treat approximately 8 million inpatients per year. According to a government survey, DPC hospitals accounted for 63% (493,581/781,022) of all acute care beds in Japan in 2014.[10] Consequently, this database is representative of more than half of all Japanese acute care hospitals.

Although DPC data mainly contain claims data for all procedures and drugs utilized during hospitalization, the data also include clinical summaries of hospitalization such as diagnoses and disease severity. Moreover, DPC data is provided in a uniform format stipulated by the government that allows the comparative analysis of many hospitals throughout Japan.

From the database, we identified hospitals that had continuously provided data throughout the study period to analyze temporal changes in LOS and readmission rates. We selected early-stage cancer surgical patients for three highly prevalent cancers as study subjects to compare changes while minimizing the influence of patient heterogeneity. For our study subjects, we identified stage I (T1N0M0) cancer surgical cases for gastric cancer (International Classification of Diseases, 10th Revision [ICD-10] code: C16.x), colon cancer (C18.x) and lung cancer (C34.x). Only patients who were discharged alive from the index hospitalization were included in analysis.

We then identified unplanned readmissions for the study subjects: 30-day unplanned emergency readmission was defined as the first readmission within 30 days after discharge from the index hospitalization for any purpose other than planned hospitalization (e.g., patient education or scheduled chemotherapy). This was determined using the indicated emergency status for hospitalization recorded in the DPC database, which distinguishes between “Emergency hospitalization” or “Other”; the latter was interpreted as indicating a planned readmission. If we detected an unplanned emergency readmission, we ascertained the clinical reason for the readmission using the reported ICD-10 codes.

We compared the temporal changes in overall, pre-operative, and post-operative LOS, as well as readmission rates across the study period. In addition, we examined LOS and the readmission rates over the years using regression models that included patient age as covariates. Statistical analyses were conducted using R software version 3.2.4. *P* values below 0.05 were considered statistically significant.

This study was approved by the Ethics Committee, Kyoto University Graduate School and Faculty of Medicine (Approval number: R0135). This study was conducted in accordance with the Ethical Guidelines for Medical and Health Research Involving Human Subjects established by the Japanese national government, which stipulates the requirements for protecting patient anonymity. Based on these guidelines, our study satisfied the necessary conditions to waive the need for informed consent, and the ethics committee approved that waiving.

## Results

We identified 804 hospitals that had continuously provided data throughout the 4-year study period. As shown in Table 1, the study subjects comprised 42,585, 15,467, and 40,156 surgical cases with gastric cancer, colon cancer, and lung cancer, respectively.

**Table 1:** Number of cases, length of stay, and 30-day unplanned emergency readmission rates in early-stage cancer surgery patients between fiscal years 2010 to 2013.

	Fiscal year	Number of cases	Mean overall LOS (days)	Mean pre-operative LOS (days)	Mean post-operative LOS (days)	Discharged directly to home	Unplanned emergency readmission within 30 days		Mean age (years)
Gastric Cancer	2010	9476	21.85	3.36	17.49	97.39%	174	(1.84%)	67.52
	2011	11539	21.55	3.25	17.30	97.33%	220	(1.91%)	68.00
	2012	11467	20.68	3.08	16.59	97.31%	192	(1.67%)	68.30
	2013	10103	19.89	2.96	15.93	97.02%	151	(1.49%)	67.97
Colon Cancer	2010	3192	18.70	3.59	14.11	97.59%	49	(1.54%)	69.35
	2011	4143	17.76	3.39	13.37	98.46%	87	(2.10%)	68.97
	2012	4212	17.75	3.31	13.44	98.17%	58	(1.38%)	69.56
	2013	3920	17.18	3.23	12.95	98.55%	58	(1.48%)	69.40
Lung Cancer	2010	8108	15.61	3.37	11.25	98.45%	126	(1.55%)	68.66
	2011	10708	15.17	3.25	10.91	98.36%	133	(1.24%)	69.01
	2012	11061	14.61	3.08	10.53	98.41%	128	(1.16%)	69.20
	2013	10279	14.25	2.88	10.38	98.35%	117	(1.14%)	69.19

LOS, length of stay

**Fig 1:**
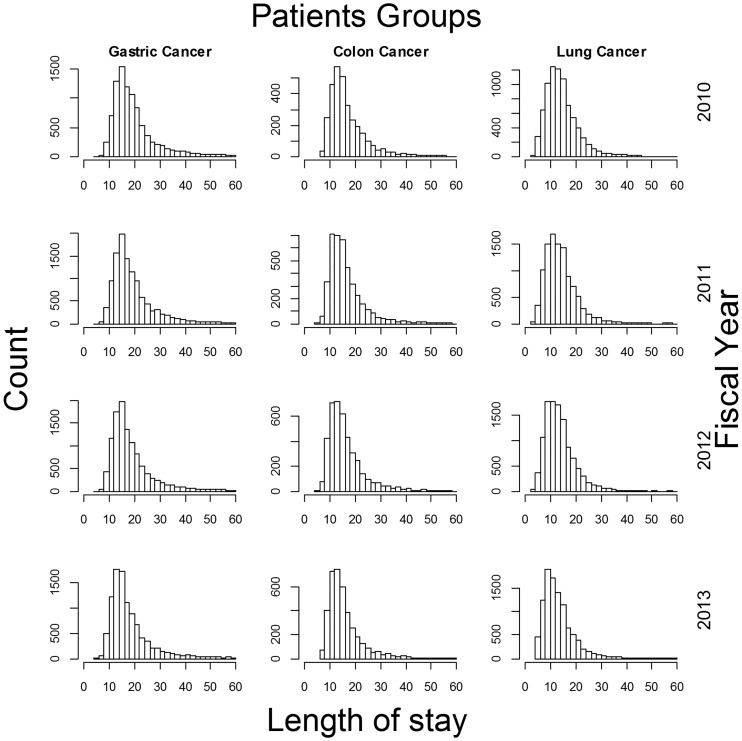
Overall length of stay in early-stage cancer surgery patients. Only cases with length of stay durations below 60 days are shown.

**Fig 2:**
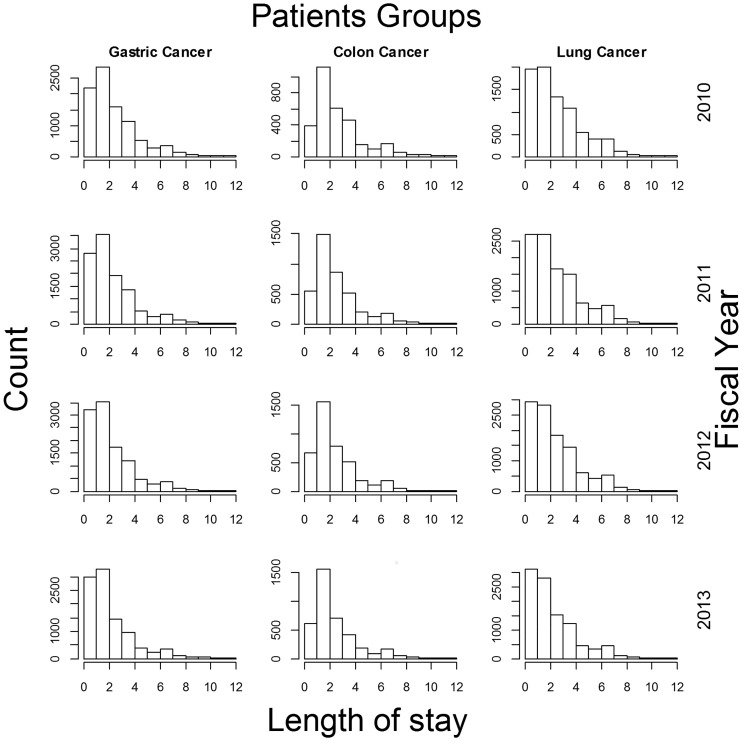
Pre-operative length of stay in early-stage cancer surgery patients. Only cases with pre-operative length of stay durations below 12 days are shown.

Overall LOS was observed to decrease over the years; similar reductions were also observed in pre-operative and post-operative LOS. The distributions of overall LOS (Fig 1) and pre-operative LOS (Fig 2) demonstrated slight reductions in spread with increases in peak height throughout the study period. More than 97% of cases were discharged directly to home, with the rates relatively stable over time. The mean age of patients was observed to increase with time (Table 1). In addition, readmission rates were mostly under 2%, and the rates were stable or had decreased slightly over the years (Table 1).

The causes of readmission are summarized according to ICD-10 codes in Table 2 (only diseases with 10 or more cases are shown). Complications of procedures (T81.x), postprocedural disorders of digestive system (K91.x), and pyothorax (J86.x) were the most common causes for unplanned emergency readmissions in gastric cancer, colon cancer, and lung cancer patients, respectively.

**Table 2:** Causes of readmission according to ICD-10 codes (only diseases with 10 or more cases are included).

	ICD-10 codes	Disease	Number of cases
Gastric Cancer	T81	Complications of procedures, not elsewhere classified	120
	K91	Postprocedural disorders of digestive system, not elsewhere classified	104
	C16	Malignant neoplasm of stomach	98
	K56	Paralytic ileus and intestinal obstruction without hernia	45
	E86	Volume depletion	35
	K65	Peritonitis	34
	J69	Pneumonitis due to solids and liquids	17
	K92	Other diseases of digestive system	16
	R63	Symptoms and signs concerning food and fluid intake	12
	J18	Pneumonia, organism unspecified	11
Colon Cancer	K91	Postprocedural disorders of digestive system, not elsewhere classified	83
	K56	Paralytic ileus and intestinal obstruction without hernia	40
	T81	Complications of procedures, not elsewhere classified	20
	C18	Malignant neoplasm of colon	11
Lung Cancer	J86	Pyothorax	54
	C34	Malignant neoplasm of bronchus and lung	39
	J93	Pneumothorax	31
	J18	Pneumonia, organism unspecified	30
	J90	Pleural effusion, not elsewhere classified	25
	J95	Postprocedural respiratory disorders, not elsewhere classified	21
	J15	Bacterial pneumonia, not elsewhere classified	21
	J84	Other interstitial pulmonary diseases	21
	T81	Complications of procedures, not elsewhere classified	21
	J98	Other respiratory disorders	16
	I50	Heart failure	14
	I63	Cerebral infarction	14

We conducted linear regression analyses of LOS to compare the time trends while accounting for patient age. The model syntax in R is as follows:

lm(los ~ age_cat + year, data=dataset)

where “los” is LOS, “age_cat” is a categorical variable for age (≤39 y, 40–64 y, 65–74 y [used as the reference category], 75–84 y, and ≥85 y), and “year” is a categorical variable for the year of the index hospitalization (reference: 2010). We conducted analyses using the same models for gastric cancer patients, colon cancer patients, and lung cancer patients. The estimated coefficients for each regression model are shown in Table 3. LOS was observed to be significantly shorter in the later years.

**Table 3:** Linear regression analysis results of length of stay for early-stage cancer patients.

	Gastric Cancer		Colon Cancer		Lung Cancer	
	Coefficients	*P* value	Coefficients	*P* value	Coefficients	*P* value
Age (years)						
≤39	-4.4425	<0.001***	-3.6334	<0.001***	-3.6589	<0.001***
40–64	-2.2167	<0.001***	-1.6276	<0.001***	-1.6772	<0.001***
65–74 (Reference)						
75–84	2.4667	<0.001***	1.486	<0.001***	0.9991	<0.001***
≥85	4.7429	<0.001***	4.3903	<0.001***	1.1692	0.0025**
Fiscal year						
2010 (Reference)						
2011	-0.3829	<0.001***	-0.8924	0.077	-0.475	0.0046**
2012	-1.3345	<0.001***	-1.0124	<0.001***	-1.0788	<0.001***
2013	-2.0889	<0.001***	-1.5713	<0.001***	-1.443	<0.001***

** *P* < 0.01,

*** *P* < 0.001

Finally, we conducted logistic regression analyses of 30-day unplanned emergency readmission to investigate the time trends while accounting for patient age. The model syntax in R is as follows:

glm(unplan_readm ~ age_cat + year, family=binomial(logit), data=dataset)

where “unplan_readm” is a binominal variable for 30-day unplanned emergency readmission, “age_cat” is a categorical variable for age (≤39 y, 40–64 y, 65–74 y [used as the reference category], 75–84 y, and ≥85 y), and “year” is a categorical variable for the year of the index hospitalization (reference: 2010). We conducted analyses using the same models for gastric cancer patients, colon cancer patients, and lung cancer patients. The estimated coefficients for each regression model are shown in Table 4. Readmission rates were observed to be significantly lower in the later years only in lung cancer patients. While analysis of the other two datasets also suggested a decrease in readmission rates, the results were not statistically significant.

**Table 4:** Logistic regression analysis results of 30-day unplanned emergency readmission for early-stage cancer patients.

	Gastric Cancer		Colon Cancer		Lung Cancer	
	Coefficients	*P* value	Coefficients	*P* value	Coefficients	*P* value
Age (years)						
≤39	-0.59316	0.153	-0.95638	0.343	-1.1875	0.237
40–64	-0.32359	0.001*	-0.22785	0.1577	-0.1604	0.191
65–74 (Reference)						
75–84	0.29404	0.001**	-0.10224	0.512	0.4507	<0.001***
≥85	0.53204	0.001**	0.0945	0.739	0.5896	0.018*
Fiscal year						
2010 (Reference)						
2011	0.09361	0.792	0.31893	0.077	-0.2366	0.059
2012	-0.02804	0.276	-0.11358	0.561	-0.3122	0.014*
2013	-0.23957	0.043*	-0.04012	0.837	-0.3255	0.012*

* *P* < 0.05,

** *P* < 0.01,

*** *P* < 0.001

## Discussion

In a large-scale analysis of Japanese acute care hospitals, we observed decreasing trends in LOS in surgical patients with early-stage gastric cancer, colon cancer, and lung cancer. However, the reduction in LOS was not accompanied by increases in readmission rates.

Overall LOS decreased gradually in all three cancer patient groups. Furthermore, both pre-operative and post-operative LOS were also observed to decrease over the 4-year study period. As the DPC/PDPS had been introduced over 10 years ago, the response of hospitals to this system may have been continual. In addition to the reductions in both pre-operative and post-operative LOS, the peak height of LOS distributions had also increased. This may indicate that hospitalizations have become more standardized. Moreover, the slightly decreasing trends in both pre-operative and post-operative LOS suggest that the planning of hospitalizations has improved due to advances in clinical pathways and medical techniques. In particular, improvements in post-operative care may have reduced LOS durations after surgery.

If this supposition is true, then the stable or decreasing readmission rates observed in this study are unsurprising. This lack of increasing readmission rates has also been reported in the existing literature, [4–6] and may imply that medical techniques or procedure choices in cancer treatment have generally improved over the years or that the DPC/PDPS has contributed to improving the efficiency and effectivity of healthcare processes. Clinical pathways are likely to have provided considerable contributions due to their systematic schemes for reviewing and planning medical care, including the establishment of standardized discharge criteria that would directly affect LOS durations. These pathways are often discussed as an integral part of the DPC/PDPS in Japan. Most of these reports are published in Japanese, and can be retrieved using the Ichushi-Web bibliographic database for Japanese medical articles (http://search.jamas.or.jp/). We conducted a search of this database on May 25, 2016 using the search terms “DPC” and “clinical pathway”. Search results were limited to original articles published within the past five years, and we excluded case reports and conference abstracts. The search retrieved 606 articles, none of which reported any worsening trends in LOS and readmission rates.

As described in this study, investigations into the association between LOS and readmission rates should be conducted using detailed analyses on specific diseases or patients. Previous analyses by the council have not examined patient cohorts that were hospitalized for specific diseases with subsequent readmission as an outcome. In contrast, we have analyzed patients with specific conditions from the index hospitalization and identified their readmissions using the database. Table 2 shows that unplanned emergency readmissions were due to a variety of causes, but the major causes for readmissions were associated with postprocedural complications. This may indicate that the use of “Emergency hospitalization” in the DPC database was generally able to identify readmissions associated with the index hospitalization, although further studies are needed to examine the clinical context and outcomes of readmissions.

Datasets can vary widely in type of diseases and severity, as well as in patient demographic factors such as age and sex. Although the reports by the MHLW detected increases in readmission rates, the wide variety in patient factors renders it difficult to interpret their findings.[2,3] Risk adjustment may help to account for these variations, and hospital standardized mortality ratios are often used as a measure of clinical outcomes. However, the use of these ratios is not without controversy, due in part to the insufficiency of adjustment variables, limitations of modeling methods, and the wide variety in causes of admissions.[11]

To reduce the effects of these limitations and variations on our results, we focused on early-stage cancer surgical patients with one of three major cancers. By restricting the study subjects to specific disease types, we are able to assume that patient heterogeneity had been reduced to facilitate comparisons within each group. In this study, we also investigated patient destination after discharge. If a patient is discharged to a care facility, a reduction in LOS would be interpreted differently than if the patient had been discharged to home. However, the rate of discharge to home was consistently above 97% throughout the study period, indicating that there were no major changes in patient destination after discharge. These detailed analyses allow for more straightforward interpretations of the data than if using whole databases comprising different diseases, severity, and patient characteristics. Moreover, our comparisons were made after accounting for patient age, which was found to be a significant determinant of 30-day unplanned emergency readmission.

On the other hand, the focus on specific diseases may also be a limitation of this study. Although we did not detect any increases in readmission rates in these diseases, the findings may not be applicable to other diseases and treatments. Further investigation is needed for other patient groups, and should also be conducted on specific diseases that take patient heterogeneity into account. While patient age had been adjusted in our study, it would also be useful to incorporate indicators of patients’ conditions such as performance status. Another limitation is that the study period only spanned 4 years. Therefore, hospitals that implemented the DPC/PDPS more recently may act differently, as noted in Hamada et al.[9] This study suggests that in the long term, the DPC/PDPS may contribute to the improvement of both LOS and readmission rates, although the system may have different short-term effects. The collection of DPC data for analyses by the government began only in July 2010, and our study was conducted using data from the start of the dataset to provide a large quantity of continuous information. We believe that the database used in this study is generally representative of acute hospitals in Japan, but investigations that cover longer study periods and include more hospitals should be conducted in the future.

## Conclusions

Although LOS for early-stage gastric, colon, and lung cancer surgical patients in Japanese acute care hospitals had decreased in recent years, there were no increases in 30-day unplanned emergency readmission. Hence, the reduction of LOS durations did not appear to detrimentally affect this aspect of health care.
